# Prediction of cardiovascular events in older patients with hypertension in primary care: a cohort study

**DOI:** 10.3399/BJGP.2023.0328

**Published:** 2024-03-28

**Authors:** Josephine ML de Hartog-Keyzer, Victor JM Pop, Laura Rodwell, Robin Nijveldt, Saloua El Messaoudi

**Affiliations:** Department of Medical and Clinical Psychology, Tilburg University, Tilburg, the Netherlands.; Department of Medical and Clinical Psychology, Tilburg University, Tilburg, the Netherlands.; Department for Health Evidence, Radboud University Medical Centre, Nijmegen, the Netherlands.; Department of Cardiology, Radboud University Medical Centre, Nijmegen, the Netherlands.; Department of Cardiology, Radboud University Medical Centre, Nijmegen, the Netherlands.

**Keywords:** brain natriuretic peptide, cardiovascular diseases, heart failure, hypertension, left ventricular hypertrophy, primary health care

## Abstract

**Background:**

Accurate risk stratification identifying patients with hypertension at risk of future cardiovascular disease in primary care would be desirable.

**Aim:**

To investigate the association between elevated brain natriuretic peptide (BNP), left ventricular hypertrophy (LVH) on an electrocardiogram (ECG), and LVH on an echocardiogram and the development of cardiovascular events (CVEs), especially heart failure and all-cause mortality (ACM), in a primary care population with hypertension without symptoms of heart failure.

**Design and setting:**

A prospective cohort study in five Dutch general practices between 2010–2012 and 2020.

**Method:**

In total, 530 patients (aged 60–85 years) underwent laboratory testing, ECGs, and echocardiograms at baseline. The incidence of new CVEs and ACM at up to 9 years’ follow-up was recorded by data extraction from the digital information systems.

**Results:**

Among the 530 participants, 31 (5.8%) developed a coronary event, 44 (8.3%) a cerebrovascular accident, 53 (10.0%) atrial fibrillation, 23 (4.3%) heart failure, and 66 (12.5%) died. Cox regression analyses, adjusting for relevant Framingham covariates, showed that elevated BNP increased the risk of ACM, CVEs, and specifically heart failure independently by 44% (hazard ratio [HR] 1.44, 95% confidence interval [CI] = 1.07 to 1.94, *P* = −0.017), 45% (HR 1.45, 95% CI = 1.15 to 1.82, *P* = 0.002), and 288% (HR 3.88, 95% CI = 2.13 to 7.10, *P*<0.001), respectively. LVH on ECG increased the risk of ACM independently by 108% (HR 2.08, 95% CI = 1.14 to 3.81, *P* = 0.017). LVH either on an ECG and/or echocardiogram increased the risk of heart failure independently by 309% (HR 4.09, 95% CI = 1.34 to 12.49, *P* = 0.014).

**Conclusion:**

In primary care patients with hypertension, BNP seems to be an important marker predicting future CVEs, especially heart failure, as well as all-cause mortality.

## Introduction

Hypertension is very common in the general population,^1^ with a prevalence of up to 62% in older adults.^2^^,^^3^ Long-term hypertension, an important cause of cardiovascular morbidity and mortality,^4^^–^^6^ requires optimal control and persistent adherence to prescribed medication to reduce the risk of cardiac, cerebrovascular, and renal disease. An important cardiac complication of poorly treated hypertension is the development of left ventricular hypertrophy (LVH), which often leads to heart failure and eventually to death.^7^^,^^8^

Accurate risk stratification identifying those at high risk for future CVE in the primary care population with hypertension would be desirable. Screening for hypertension-mediated organ damage is crucial, as highlighted by the World Health Organization.^9^ The presence of LVH on an electrocardiogram (ECG) has been shown to be of prognostic value, but the sensitivity and specificity of ECGs to detect LVH is limited. Therefore, echocardiography has become the routine gold-standard test to detect LVH.^10^^,^^11^ There is increasing interest in the potential use of measuring the blood level of brain natriuretic peptide (BNP) for clinical stratification of cardiovascular risk in patients with hypertension.^12^ Primary care physicians play a critical role in the detection, risk stratification, and treatment of patients with hypertension. The current European and UK GP guidelines for the management of hypertension do recommend an ECG at the start of treatment but no other additional diagnostic tests, like echocardiography and/or the BNP assessment.^5^^,^^13^ The Dutch guidelines do not recommend any routine investigation at diagnosis of hypertension.^14^

It is conceivable that, even in patients with hypertension without symptoms of heart failure, BNP assessment, an ECG, and echocardiography may be valuable in predicting future cardiovascular disease (CVD). The authors of the current study have previously found a high overall prevalence of previously undetected LVH using ECGs and echocardiography in 44% of a primary care population with hypertension.^15^ It is currently unclear whether these patients are more likely to develop a cardiovascular event and/or have a higher mortality risk.

**Table:** How this fits in

Accurate risk stratification identifying those at high risk for future cardiovascular events in the primary care population with hypertension would be desirable. Notably, primary care guidelines advocate an electrocardiogram (ECG) at hypertension onset, but no routine echocardiography or brain natriuretic peptide (BNP) assessment. This primary care study of older patients with uncomplicated hypertension shows that BNP analysis has an important predictive value for all-cause mortality and the occurrence of CVEs, in particular heart failure, and, moreover, seems to be a stronger predictor compared with an ECG and echocardiogram. BNP analysis should therefore be considered as an additional diagnostic tool to the standard risk stratification.

The main objective of the current study was therefore to investigate the predictive value of LVH on an ECG and/or on echocardiography and/or an elevated BNP, for the development of future cardiovascular events (CVEs) and all-cause mortality (ACM) in primary care patients with hypertension without symptoms of heart failure. The prospective design enabled follow-up data up to 9 years.

## Method

### Participants

Patients in the current study were enrolled in the CHELLO study (Chronic Heart Failure Prevention Program), the details of which have already been published.^15^^–^^19^ In summary, between June 2010 and January 2012, a total of 913 primary care patients, aged 60–85 years, with an International Classification of Primary Care (ICPC) code for hypertension (K86/K87) were invited to participate in the CHELLO study.

Inclusion criteria were: older primary care patients (aged 60–85 years) with hypertension included in the primary care cardiovascular risk management programme, without a diagnosis of heart failure (ICPC K77). Patients with a history of CVD (for example, myocardial infarction) were only included if they were no longer treated by a cardiologist. A total of 595 eligible patients gave written informed consent for participation.

### Study procedure and data collection

After obtaining written informed consent, a healthcare nurse conducted a structured interview and performed an examination focusing on blood pressure, weight, and height assessment during a baseline visit. After 20 min of rest in a sitting position, blood pressure was automatically measured three times in a row with a digital blood pressure monitor with an upper cuff (first mean blood pressure) and was repeated after 20 min (second mean blood pressure). The mean value of both procedure outcomes was defined as the systolic/diastolic blood pressure, which was used for data analyses.

In addition, demographic and clinical variables were obtained during the interview and after reviewing the patient’s medical records. Following the first appointment, blood was drawn by venepuncture to measure BNP and lipid profile.

A follow-up visit was planned at the patient’s primary care practice to perform an ECG and echocardiogram. The ECG and echocardiogram were carried out by a trained, experienced echocardiographer from ‘Diagnostiek voor U’ in Eindhoven, the Netherlands, a primary care laboratory and medical diagnostics organisation.

A standard resting 12-lead ECG was recorded (paper speed 25 mm/s, 10 mm/mV). In the current study, the eight well-known and often-used criteria (Supplementary Table S1) were used to evaluate LVH features during ECG.^20^^–^^25^ If ≥1 of these eight criteria were positive, ECG–LVH was diagnosed. An independent cardiologist who was blinded for outcomes of the echocardiogram gave a final review.

A transthoracic 2D echocardiographic examination was performed with an s5 transducer (Philips CX 50) in a standard position. All of the echocardiograms were reviewed by a panel of cardiologists specialised in echocardiography, according to European recommendations and guidelines for evaluating chamber quantification, diastolic dysfunction, and heart valve disease.^26^^–^^28^ LVH was defined as any abnormal left ventricular size measurement (septal or posterior wall thickness of >0.9 cm in females or >1.0 cm in males) and calculated left ventricular mass index adjusted for body surface area >95 g/m^2^ in females and >115 g/m^2^ in males. The Cube formula for LV mass was used:^27^

$$0.8\times 1.04\times [{\left(\text{IVS}+\text{LVID}+\text{PWT}\right)}^{3}-{\text{LVID}}^{3}]+0.6\text{g}$$

where IVS is the interventricular septum; LVID is the left ventricular internal diameter; and PWT is the posterior (inferolateral) wall thickness.

### Definition of the groups

To answer the study’s research question, different groups were defined relating to the different tests:

- Group 1 distinguished between those with normal and those with elevated BNP. A cut-off of ≥10 pmol/L was used, which corresponds to ≥35 pg/mL, in the European and Dutch guidelines.^29^^,^^30^
- Group 2 distinguished between the presence and absence of LVH on the ECG, according to the criteria described above.
- Group 3 were patients with or without LVH on the echocardiogram.
- Group 4 distinguished between the presence and absence of LVH on either the ECG and/or the echocardiogram.

### The incidence of new CVEs and ACM during follow-up

To evaluate the incidence of new CVEs and ACM, a data extraction of the digital GP information system was performed in January 2020, providing follow-up data up to 9 years. Every GP in the Netherlands uses standard diagnosis codes for data entry into the electronic patient record form: ICPC. The occurrence of the following CVDs and deaths was registered: the development of coronary events (acute coronary syndrome; unstable angina pectoris or acute myocardial infarction), heart failure, atrial fibrillation, cerebrovascular diseases (transient ischaemic attack and cerebrovascular accident), and death with the ICPC codes: K74, K75, K76, K77, K78, K89, K90, and A96, respectively.

### Statistical analyses

For the baseline characteristics, categorial variables are shown as numbers (percentage), continuous variables as mean (with standard deviation [SD]), and non-normally distributed variables as median (with interquartile range [IQR]). For the comparison of the incidence of new CVEs and ACM during follow-up between males and females, the χ^2^-test was used. Cumulative incidence curves for the outcomes of ACM, all CVEs together, and heart failure were plotted using the ggsurvfit package in R.^31^ Curves were plotted for patients, using the four groups as described above, using years of follow-up as the timescale.

Univariable and multivariable Cox regression analyses (hazard ratios [HRs] and 95% confidence intervals [CIs]) were used to estimate the relationship between an elevated BNP level, LVH on an ECG, and LVH on an echocardiogram and developing a (specific) cardiovascular event or death. BNP level was included in the models as a log-transformed and continuous variable. Baseline Framingham risk variables included were used as potential confounders (age, gender, smoking, cholesterol, and systolic blood pressure).

All data were analysed with commercially available statistical software (IBM SPSS Statistics version 26) and with R.

## Results

### Study population

At baseline, a total of 592 patients provided written informed consent for participation in the CHELLO study.^15^^–^^19^ For the current study, 23 patients were excluded based on missing baseline data (ECG and/or echocardiogram). Five patients were excluded based on poor image quality of the echocardiogram. At 9 years, of the remaining 564 participants, 34 patients were lost to follow-up. Baseline characteristics of patients who dropped out were similar to those who completed study duration. Therefore, data analyses in the current study included a total of 530 patients.

### Baseline characteristics

Baseline demographics of the patient cohort are displayed in Table 1. The mean age was 70 years (SD 6.5) and 56.8% (*n* = 301/530) of the study population were female. At baseline, 257 (48.5%) patients had an elevated BNP (≥10 pmol/l), 59 (11.1%) had LVH on ECG, and 190 (35.8%) had LVH on echocardiogram.

**Table 1.** Baseline demographics of 530 primary care patients with hypertension with 9 years’ follow-up

**Characteristic**	***n* (%)^a^**
**Demographics**	
Age, mean (SD)	70 (6.5)
Female	301 (56.8)
Male	229 (43.2)

**Socioeconomics**	
Have a partner	398 (75.1)
Low education^b^	72 (13.6)

**Lifestyle**	
Current smoker	67 (12.6)
Regular alcohol use^c^	165 (31.1)
Recommended physical exercise^d^	78 (14.7)

**Clinical characteristics and risk factors**	
Previous myocardial infarction	27 (5.1)
Previous CVA (TIA/stroke)	49 (9.2)
Diabetes	59 (11.1)
Peripheral artery disease	19 (3.6)
BMI, mean (SD)	28 (4.5)
SBP, mean (SD)	150 (19.6)
SBP >140	356 (67.2)
SBP >150	233 (44.0)
DBP, mean (SD)	82 (10.5)
DBP >90	101 (19.1)
Blood pressure on target^e^	212 (40.0)
Use of antihypertensive medication	407 (76.8)
Years of hypertension, median (IQR)	10.0 (4.0–16.0)
Total cholesterol, mean (SD)	5.1 (1.0)
LDL, mean (SD)	3.1 (0.9)
LDL on target^f^	128 (24.2)
Use of cholesterol-lowering medication	233 (44.0)
BNP, median (IQR)	10.0 (5.7–18.0)
Elevated BNP ≥10 pmol/l	257 (48.5)
LVH on ECG	59 (11.1)
LVH on echocardiogram	190 (35.8)

a *Unless otherwise stated.*

b *Only primary elementary educated.*

c *Defined as ≥2 glasses of alcohol per day on average.*

d *Defined as ≥30 min exercise per day, at least 5 days per week.*

e *On-target SBP defined as <140 mmHg for people aged <70 years and <150 mmHg for people aged ≥70 years, according to Dutch primary care guideline.^14^*

f *On-target LDL defined as <1.8 mmol/l for people aged <70 years and previous CVD, <2.6 mmol/l for people aged <70 years without previous CVD, and <2.6 mmol/l for people aged ≥70 years, according to the Dutch primary care guideline.^14^ BMI = body mass index. BNP = brain natriuretic peptide. CVA = cerebrovascular accident. CVD = cardiovascular disease. DBP = diastolic blood pressure. ECG = electrocardiogram. IQR = interquartile range. LDL = low-density lipoprotein. LVH = left ventricular hypertrophy. SBP = systolic blood pressure. SD = standard deviation. TIA = transient ischaemic attack.*

The characteristics of the current sample did not differ from the original CHELLO cohort,^16^^–^^18^ and patients who were lost to follow-up did not differ based on age and gender.^19^

### CVEs and ACM during 9 years’ follow-up

The date of the follow-up extraction was 2 January 2020. The median duration of follow-up was 96.0 months (IQR 89.0–105.3).

Table 2 shows the incidence of all new CVEs (*n* = 126) and ACM (*n* = 66) during 9 years’ follow-up. Of the participants, 31 (5.8%) had a coronary event, 44 (8.3%) a cerebrovascular event, 53 (10.0%) atrial fibrillation, and 23 (4.3%) developed heart failure. Of all patients, 27 (5.1%) had >1 CVE during the follow-up period.

**Table 2.** Incidence of new cardiovascular events and death at 9 years’ follow-up

**Cardiovascular event and death**	**Total, *n* (%), *n* = 530**	**Male, *n* (%), *n* = 229**	**Female, *n* (%), *n* = 301**	***P*-value**
**Coronary event**	31 (5.8)	15 (6.6)	16 (5.3)	0.549
Myocardial infarction	9 (1.7)	2 (0.9)	7 (2.3)	0.200
Unstable angina pectoris	22 (4.2)	13 (5.7)	9 (3.0)	0.125

**Cerebrovascular disease**	44 (8.3)	20 (8.7)	24 (8.0)	0.753
TIA	22 (4.2)	9 (3.9)	13 (4.3)	0.824
Stroke	24 (4.5)	11 (4.8)	13 (4.3)	0.790

**Atrial fibrillation**	53 (10.0)	28 (12.2)	25 (8.3)	0.136

**Heart failure**	23 (4.3)	8 (3.5)	15 (5.0)	0.404

**All-cause mortality**	66 (12.5)	32 (14.0)	34 (11.3)	0.355

**CVE, 1**	126 (23.8)	58 (25.3)	68 (22.6)	0.464

**CVE, >1**	27 (5.1)	14 (6.1)	13 (4.3)	0.352

*CVE = cardiovascular event. TIA = transient ischaemic attack.*

Of the 126 patients with a new CVE, at baseline, 77 had an elevated BNP (61.1%), 19 had LVH on ECG (15.1%), and 46 had LVH on echocardiogram (36.5%). When the authors only focused on the 23 patients who developed heart failure these numbers were 23 (100%) for elevated BNP, five (21.7%) for LVH on ECG, and 13 (56.5%) for LVH on echocardiogram (data not shown).

No differences were found between males and females in the occurrence of overall CVEs or ACM (Table 2).

### Diagnostic tests in relation to CVEs, heart failure, and ACM

**Figure 1.**
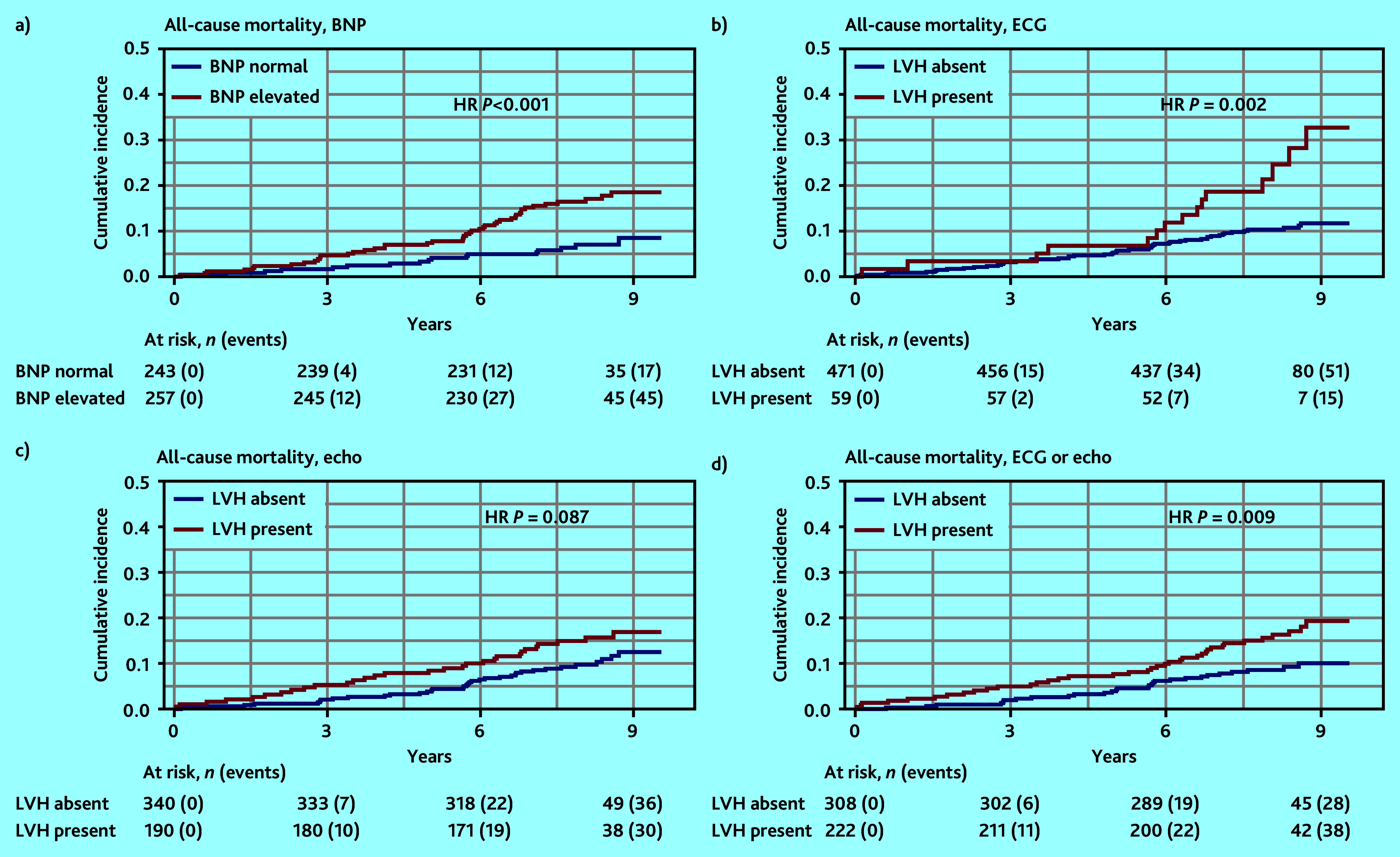
Cumulative incidence curves of all-cause mortality with diagnostic tests: a) BNP; b) ECG; c) echocardiography; and d) ECG or echocardiography. BNP = brain natriuretic peptide. ECG = electrocardiogram. Echo = echocardiography. HR = hazard ratio. LVH = left ventricular hypertrophy.

**Figure 2.**
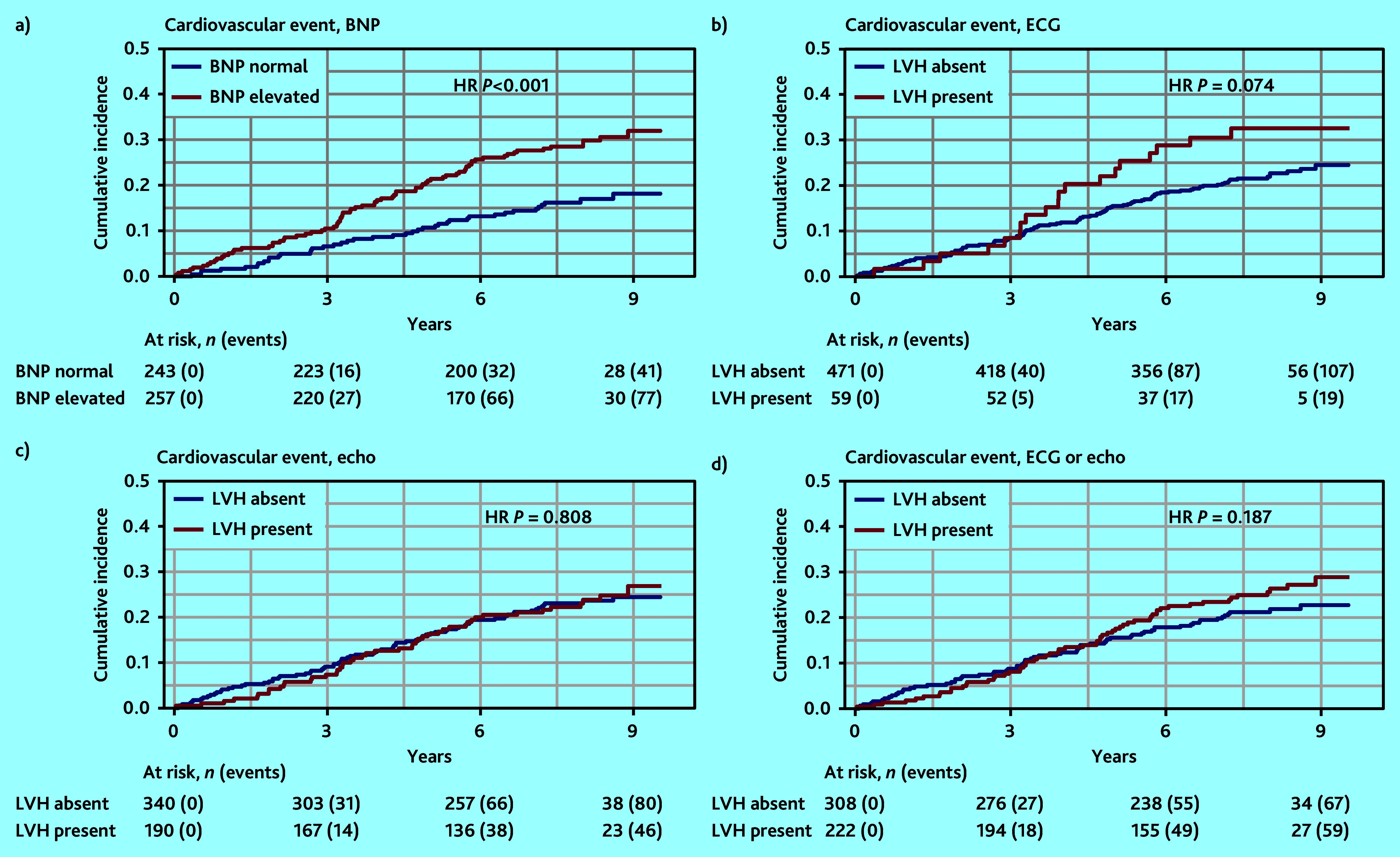
Cumulative incidence curves of cardiovascular events with diagnostic tests: a) BNP; b) ECG; c) echocardiography; and d) ECG or echocardiography. BNP = brain natriuretic peptide. ECG = electrocardiogram. Echo = echocardiography. HR = hazard ratio. LVH = left ventricular hypertrophy.

**Figure 3.**
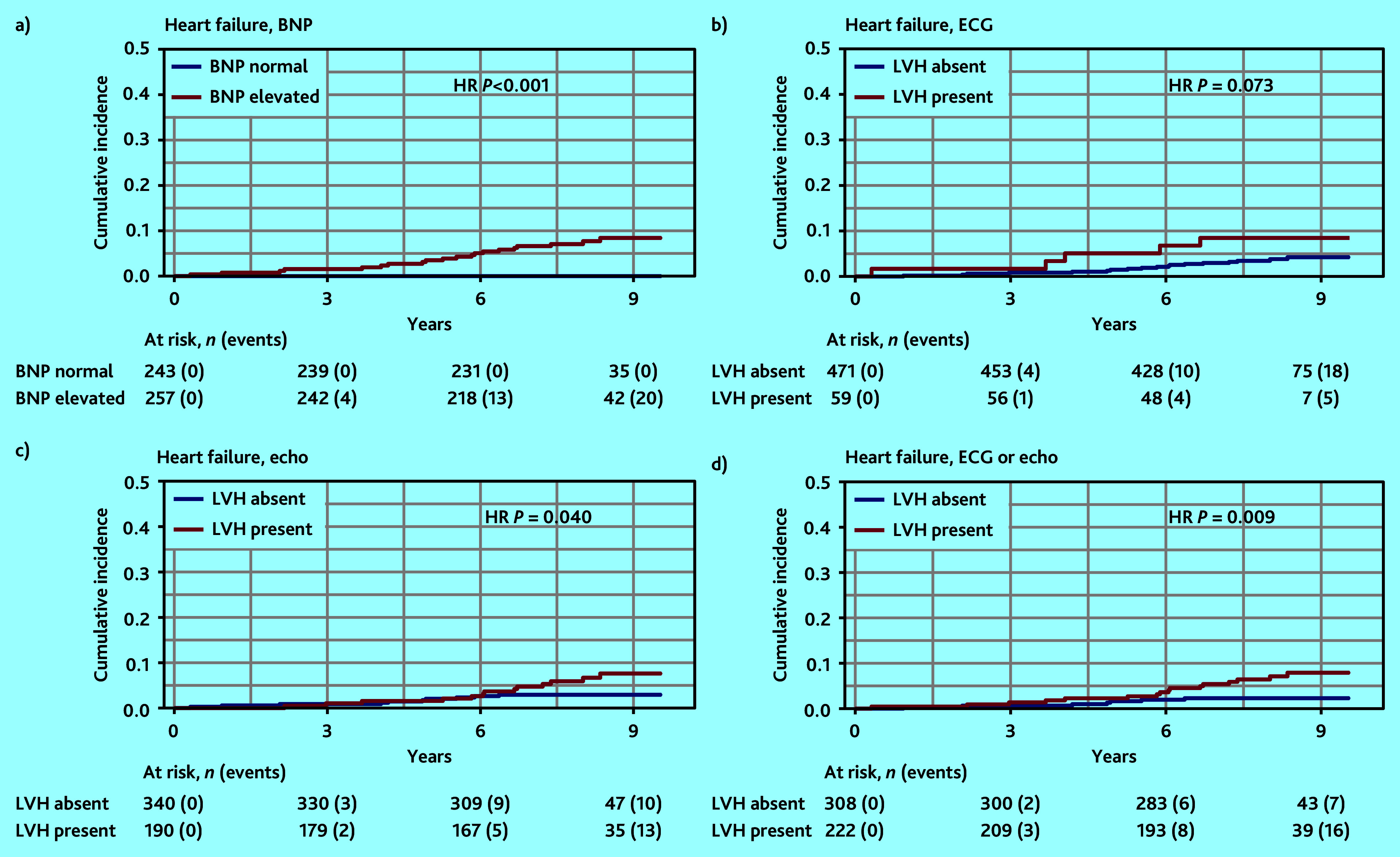
Cumulative incidence curves of heart failure with diagnostic tests: a) BNP; b) ECG; c) echocardiography; and d) ECG and echocardiography. BNP = brain natriuretic peptide. ECG = electrocardiogram. Echo = echocardiography. HR = hazard ratio. LVH = left ventricular hypertrophy.

Cumulative incidence curves, Figures 1–3, show substantial differences in the direction and strength of the associations between each of the endpoints (that is, ACM, CVE, and heart failure) and a positive diagnostic test (that is, elevated BNP, LVH on ECG, LVH on echocardiogram, and LVH on ECG and/or echocardiography). The difference in the cumulative incidence of ACM (Figure 1) in patients with or without elevated BNP and with or without LVH on ECG was the largest (HR 2.07, 95% CI = 1.58 to 2.70, *P*<0.001 and HR 2.49, 95% CI = 1.40 to 4.43, *P* = 0.002, respectively).

The cumulative incidence of CVEs (Figure 2) in patients with or without elevated BNP shows the largest difference (HR 1.60, 95% CI = 1.30 to 1.95, *P*<0.001).

The largest difference in the cumulative incidence of heart failure (Figure 3) was seen between patients with or without elevated BNP and with or without LVH either on ECG and/or echo (HR 3.98, 95% CI = 2.52 to 6.30, *P*<0.001 and HR 3.29, 95% CI = 1.35 to 7.99, *P* = 0.009, respectively).

**Figure 4.**
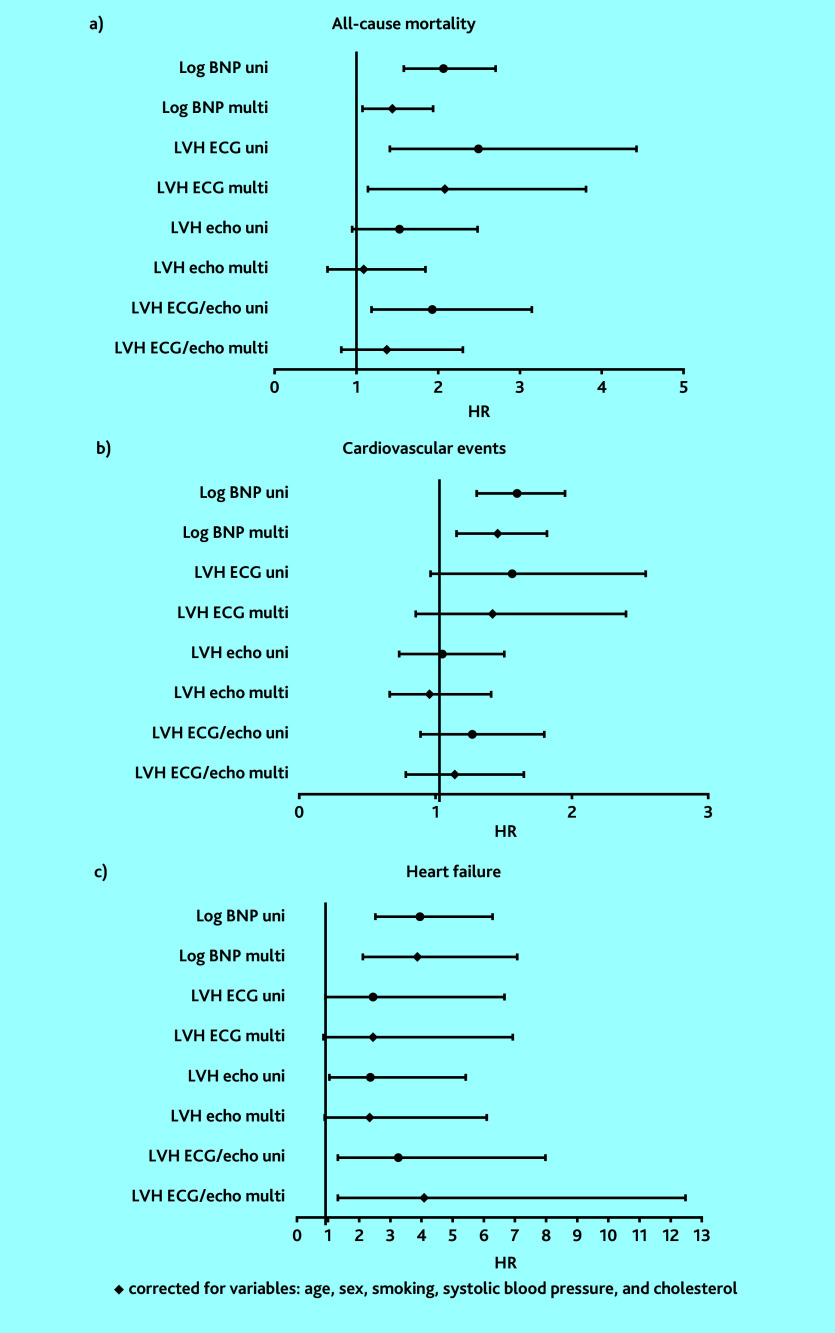
Forest plot of HRs and 95% CIs for different diagnostic tests in relation with a) all-cause mortality; b) cardiovascular events; and c) heart failure. BNP = brain natriuretic peptide. ECG = electrocardiogram. Echo = echocardiography. HR = hazard ratio. LVH = left ventricular hypertrophy. Multi = multivariate. Uni = univariate.

Figure 4 shows the HRs, using multivariable Cox regression analyses after adjustment for the baseline risk variables (age, gender, smoking, cholesterol, and systolic blood pressure). Supplementary Table S2 shows specifications of the HRs. Elevated BNP (Group 1) was still significantly associated with the occurrence of ACM, CVEs, and heart failure (HR 1.44, 95% CI = 1.07 to 1.94, *P* = 0.017; HR 1.45, 95% CI = 1.15 to 1.82, *P* = 0.002; and HR 3.88, 95% CI = 2.13 to 7.10, *P*<0.001, respectively). LVH on ECG (Group 2) was still significantly associated with ACM (HR 2.08, 95% CI = 1.14 to 3.81, *P* = 0.017). LVH either on ECG and/or echocardiography (Group 4) was still significantly associated with heart failure (HR 4.09, 95% CI = 1.34 to 12.49, *P* = 0.014).

## Discussion

### Summary

In a random sample of primary care patients with hypertension but without symptoms of heart failure the current study found a significant and independent association between elevated BNP at baseline and the occurrence of ACM and new CVEs, specifically heart failure, after 9 years’ follow-up. Presence of LVH on ECG at baseline was only significantly and independently associated with ACM. Presence of LVH on ECG and/or echocardiography at baseline was only significantly and independently associated with the occurrence of heart failure, however; interestingly, with a less strong predictive value than BNP.

### Strengths and limitations

One strength of the current study is the relatively large primary care sample with carefully registered follow-up data up to 9 years. This enabled the authors to evaluate possible associations between different diagnostic tests and the development of ACM, new CVEs, and, in particular, heart failure.

A limitation is that the outcome of the diagnostic tests used at baseline could have potentially affected the treatment of hypertension. It is conceivable that finding an elevated BNP or LVH on ECG and/or echocardiogram led to more intensive treatment. However, more intensive treatment could theoretically only have attenuated the association between elevated BNP at baseline and the occurrence of heart failure, CVEs, and ACM.

Another limitation is that the levels of BNP at baseline could possibly be influenced by medication (use of diuretics or angiotensin-converting enzyme [ACE] inhibitors), potentially decreasing BNP production.^32^ Conversely, conditions like chronic obstructive pulmonary disease (COPD) or diabetes can elevate BNP levels.^33^^,^^34^ However, in this study, BNP levels showed no difference between diuretics/ACE inhibitor users and non-users, and between people with and without diabetes. Unfortunately, COPD and cirrhosis data were unavailable in this cohort.

Furthermore, a limitation of the study was that only data on ACM and not on cardiovascular death specifically were available, a result of ethical limitations regarding the privacy of centrally registered mortality causes. Therefore, it was not possible to come to a conclusion about any association between the diagnostic tests and possible cardiovascular death.

### Comparison with existing literature

In the current study population, 24% of patients developed a CVE during 9 years of follow-up and 4.3% of patients developed heart failure. These results are in line with findings in the literature:^35^ in a primary care population in the UK, the prevalence of CVEs increased with age, with a prevalence up to 25% in those aged 70–79 years. Additionally, this UK study showed a prevalence of heart failure of 1.1% in the total population, but they did not stratify by age category. In 2019, the prevalence of registered heart failure in the Dutch population aged ≥65 years was 6.1%.^29^

The predictive value of BNP for the occurrence of heart failure, CVEs, and death is mainly investigated in high-risk populations,^36^ for example, in patients with diabetes mellitus.^37^^,^^38^ Literature on the relationship between BNP, CVEs, and death in primary care patients without symptoms is scarce.^39^^–^^41^ The studies undertaken in this specific primary care population have shown that the additional use of BNP as a risk modulator is complementary compared with the use of traditional risk factors only.^38^^–^^40^ This finding might be explained by the fact that an elevated BNP is an actual marker of elevated filling pressure in the heart, because of intra- or extra-cardiac factors.^36^

### Implications for research and practice

In the current European^5^ and UK^13^ primary care guidelines for cardiovascular risk management, an initial ECG for evidence of LVH at diagnosis of hypertension is advocated. The current study suggests that an additional BNP assessment, even in patients with hypertension without symptoms of heart failure, is useful in detecting patients at risk for ACM, overall CVEs, and, more specifically, the occurrence of heart failure.

According to the QRISK3 classification system,^42^ all patients in the current cohort were already at high risk of developing CVE by virtue of age and hypertension. Within this high-risk group there is a subgroup that are at a very-high risk for future CVEs: aged >60 years with an increased BNP.

In those patients with an increased BNP, GPs should be careful prescribing negative inotropic agents (such as calcium antagonists) for treatment of hypertension and focus on ACE inhibitors or angiotensin receptor blockers, which have demonstrated efficacy in heart failure prevention.^30^ Moreover, high-risk patients with hypertension should receive more aggressive treatment strategies to achieve target blood pressures.^39^

Future research should inform guideline committees on the necessity of periodic BNP assessments (like annual glucose and cholesterol assessments). BNP laboratory testing is easily accessible for primary care physicians, relatively inexpensive, and demonstrates strong predictive power for future CVEs. The current findings suggest that in older patients with hypertension, an ECG is less sensitive for predicting future CVEs and mortality compared with BNP assessment.

After determination of the BNP level, an echocardiogram seems no longer required in primary care as a standard examination to estimate cardiovascular risk. Echocardiography should only be considered if significant heart murmurs are present or BNP levels appear to be substantially elevated, and will help to decide whether a consultation with a cardiologist is indicated. Given the ageing population, cost reduction in health care is imperative.

In conclusion, the current primary care study of older patients with hypertension shows that BNP analysis has an important predictive value for ACM and the occurrence of CVEs, in particular heart failure. BNP analysis should therefore be considered as an additional diagnostic tool to standard risk stratification.

## Data

Data are unavailable.
